# Supplementary material for: Antiviral Wolbachia strains associate with Aedes aegypti endoplasmic reticulum membranes and induce lipid droplet formation to restrict dengue virus replication
Source: mBio. 2023 Dec 22;15(2):e02495-23. doi: 10.1128/mbio.02495-23 (PMC10865983; doi:10.1128/mbio.02495-23)
Supplement: Supplemental material — Supplemental text, figures, movie legend, and references. [file mbio.02495-23-s0001.docx]

**Supplementary information for:**

**Antiviral *Wolbachia* strains associate with *Aedes aegypti* endoplasmic reticulum membranes and induce lipid droplet formation to restrict dengue virus replication**

Robson K. Loterio^1^, Ebony A. Monson^2*^, Rachel Templin^3*^, Jyotika T. Bruyne^4^, Heather A. Flores^5^ Jason M. Mackenzie^6^, Georg Ramm^3^, Karla J. Helbig^2^, Cameron P. Simmons^1,4^, Johanna E. Fraser^1#^

^1^Department of Microbiology, Biomedicine Discovery Institute, Monash University, Clayton, Australia; ^2^Department of Physiology, Anatomy, and Microbiology, School of Life Sciences, La Trobe University, Melbourne, Australia; ^3^Ramaciotti Centre For Cryo-Electron Microscopy, Monash University, Clayton, Australia; ^4^World Mosquito Program, Monash University, Clayton, Australia; ^5^School of Biological Sciences, Monash University, Clayton, Australia; ^6^Department of Microbiology and Immunology, University of Melbourne at the Peter Doherty Institute for Infection and Immunity, Melbourne, Australia. *These authors contributed equally.

# Corresponding author: johanna.fraser@monash.edu

**This PDF file includes:**

Supplementary text information

Supplementary Fig. 1 to Fig.3

Supplementary Movie 1 legend

SI References

**Supplementary text information:**

**Extended Material and Methods**

**Mosquito rearing**

All *Ae*. *aegypti* mosquitoes were reared and maintained as described previously ^1^. Briefly, adult mosquitoes were maintained at 26˚C, 65% relative humidity, and a 12 h light:dark cycle in a climate-controlled room, and were allowed access to 10% sucrose *ad libitum.* Mosquitoes were blood fed on the arms of human volunteers for reproduction. To generate a panel of genetically comparable *Wolbachia*-carrying *Ae*. *aegypti* lines, we backcrossed females from the *w*Mel, *w*AlbB, and *w*Pip to males of the inbred laboratory *Ae*. *aegypti* line, Rockefeller ^2^ (BEI resources), for six generations. To exclude any influence of mosquito age on our experiments, age-controlled adults emerging within a 48 hours window were used.

**Live Lipid Droplet Staining**

For live staining of LDs, *Wolbachia*-free and *w*Mel-*Aag2* cell lines were seeded in a 96-well black cell culture plate with 6 replicates per condition and at 1 x 10^5^ cells/well. Cells were incubated for 24 hours at 26 °C with *Aag2* cell culture medium with 10% FBS. After 24 hours, the culture medium was replaced with fresh medium containing DMSO only, 1 µM C75, 2.5 µM DGAT 1 (T863), 10 µM DGAT 2 (PF-06424439) or DGAT 1 and 2 combined, and incubated for another 24 hours. After incubation time, cell culture medium was replaced with 5 µM BODIPY 493/503 in cell culture medium for 1 hour at 26 °C. For each condition, extra wells were designated to not have cells or cells without BODIPY staining for appropriate background reduction. Cells were then gently washed 2 times with warm PBS and cells were maintained in PBS for fluorescence acquisition. Fluorescence was detected with a microplate reader (BioTek Gen5 - Agilent, Santa Clara, CA, USA) equipped for excitation in the 485/20 nm range and emission detection at 535/20 nm (FITC wavelength).

**
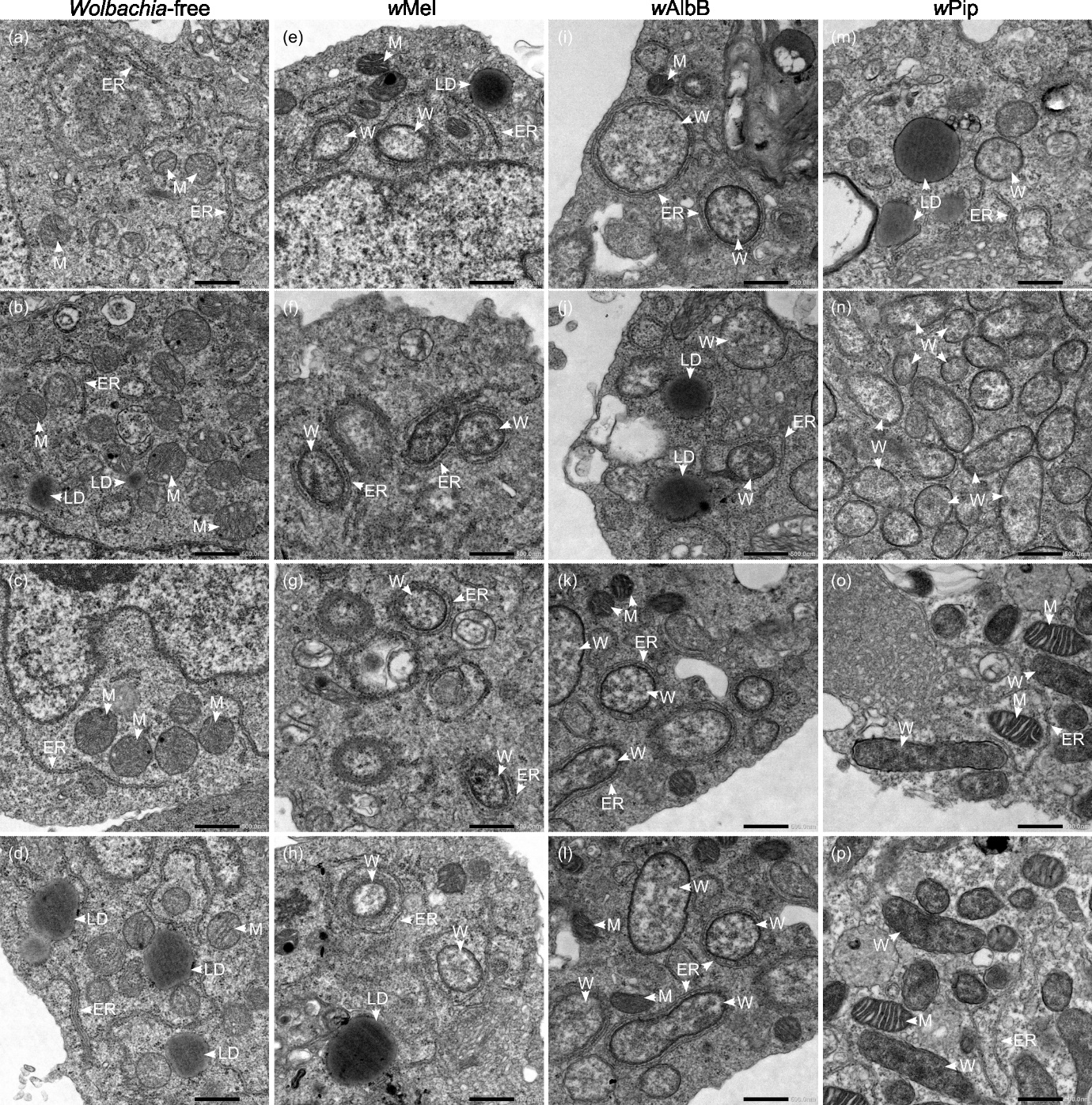
**

**Supplementary Fig. 1: Antiviral *Wolbachia* strains are frequently wrapped by the host endoplasmic reticulum membranes.** TEM micrographs of *Wolbachia*-free-*Aag2* cell line (a-d) or stably infected with *w*Mel (e-h), *w*AlbB (i-l), and *w*Pip (m-p) show their intracellular distribution and association with the ER membranes. Scale bar = 500nm. ER- Endoplasmic reticulum. W- *Wolbachia*. M- Mitochondria. LD- Lipid Droplet.


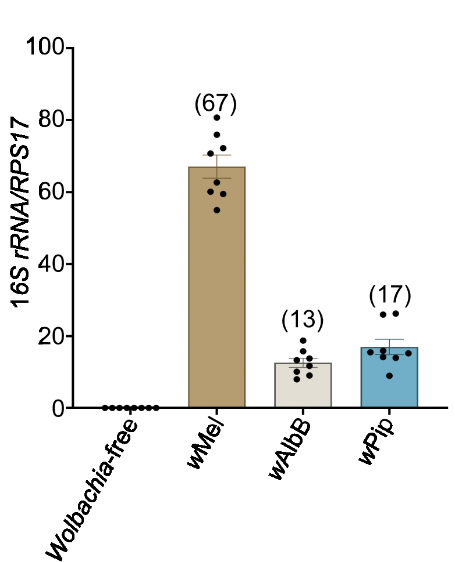


**Supplementary Fig. 2: *Wolbachia* density in *Ae. aegypti* ovaries.** Ovaries of at least 8 non-blood-fed female mosquitoes of Rockefeller (*Wolbachia*-free), Rockefeller-*w*Mel, Rockefeller-*w*AlbB, and Rockefeller-*w*Pip mosquito lines were dissected. *Wolbachia* per cell (in parentheses) was determined by qPCR (*Wolbachia* *16S rRNA/RPS17*). Data are mean with ± Standard Error of the Mean (±SEM).

**
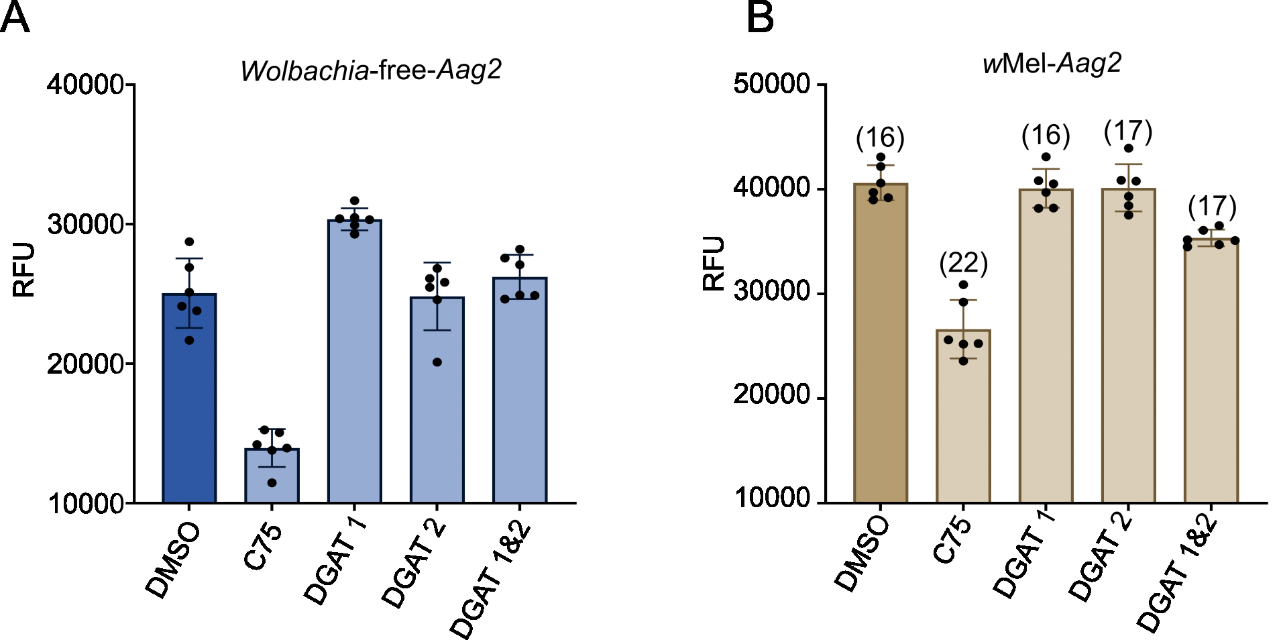
**

**Supplementary Fig. 3: The Fatty Acid Synthase (FAS) inhibitor, C75, efficiently reduces LD accumulation in *Aag2* cell lines.** (A) *Wolbachia*-free and (B) *w*Mel-*Aag2* cell lines were treated with C75, DGAT 1, DGAT 2, and DGAT 1&2 combined for 24 hours prior to LD staining. For live staining of LDs, cells were incubated with BODIPY 493/503 for 1 hour. The numbers in parentheses represent the average number of *Wolbachia* per cell (*Wolbachia* *16S rRNA*/*RPS17*) after each treatment. Data are mean with ± Standard Deviation (SD) and represent 1 independent experiment with six biological replicates each. Fluorescence was detected with a microplate reader at 485/535 nm (excitation/emission) range. RFU – Relative fluorescence units.

**Supplementary Movie 1 legend:**

200nm sections of *w*Mel-Aag2 cells were collected on copper mesh grids for electron tomography. 10nm gold fiducials were added to both sides of the section. Single axis tomography was performed on a Jeol JEM1400-Plus at 120kV using Jeol Recorder software. Tilt series were recorded with tilt angles from + 65° to − 65° with varying tilt increments based on a Saxton scheme. IMOD software 99 was used to reconstruct tilt series (etomo package), manual segmentation and visualization. ER membranes are shown in yellow, *w*Mel is indicated in blue.

**SI References**

1. McMeniman CJL, Roxanna V.; Cass, Bodil N.; Fong, Amy W.C.;, Sidhu MW, Yu-Feng; Scott L., O’Neill. . Stable Introduction of a Life-Shortening Wolbachia Infection into the Mosquito Aedes aegypti. *Science* **323**, (2009).

2. Kuno G. Early history of laboratory breeding of Aedes aegypti (Diptera: Culicidae) focusing on the origins and use of selected strains. *J Med Entomol* **47**, 957-971 (2010).
